# Unsichtbar in der fachkulturellen Erinnerung der Medizin? Die als jüdisch verfolgte Dresdener Urologin und Venerologin Dora Gerson (1884–1941)

**DOI:** 10.1007/s00120-025-02614-5

**Published:** 2025-06-06

**Authors:** Julia Nebe, Matthis Krischel

**Affiliations:** Institut für Geschichte, Theorie und Ethik der Medizin, Centre for Health and Society, Medizinische Fakultät, Heinrich-Heine-Universität Düsseldorf, Düsseldorf, Deutschland

**Keywords:** Geschichte der Medizin, Nationalsozialismus, Suizid, Frauen, Erinnerungskultur, History of medicine, National Socialism, Suicide, Women, Cultures of remembrance

## Abstract

Die Fachgeschichte der Urologie gilt traditionell als männlich geprägt. Dabei ist das Fach nie ausschließlich „Männerheilkunde“ gewesen – weder in Bezug auf die Patientenschaft noch das medizinische Personal. Der Beitrag beleuchtet exemplarisch das Leben und Wirken der Dresdner Ärztin Dora Gerson (1884–1941), einer der ersten deutschen Fachärztinnen für Urologie und Dermatovenerologie und macht auf die weitgehende Unsichtbarkeit von Frauen im fachkulturellen Gedächtnis aufmerksam. Gerson studierte zu Beginn des 20. Jahrhunderts Medizin in München und Leipzig und war in den Folgejahren sowohl klinisch als auch sozialmedizinisch tätig. In ihrer Dresdner Praxis vereinte sie Urologie, Dermatologie und Venerologie und leitete zugleich eine öffentliche Beratungsstelle für geschlechtskranke Frauen. 1933 wurde Gerson die Kassenzulassung entzogen, sie musste ihre Praxis schließen und war ab 1940 als jüdische „Krankenbehandlerin“ an der Gartenbauschule in Ahlem in Hannover tätig. Im September 1941 nahm sie sich unter dem Druck zunehmender Repressionen das Leben. Ihre Biographie steht für eine doppelte Marginalisierung: als Angehörige eines strukturell benachteiligten Geschlechts und als Opfer nationalsozialistischer Verfolgung. Der Beitrag verknüpft Gersons Lebensweg mit Fragen nach Geschlecht, Erinnerungskultur und Anerkennungspraxis in der Medizin. Er zeigt auf, wie fachkulturelles Gedächtnis selektiv funktioniert und welchen Einfluss soziale Herkunft, Geschlecht und politische Umstände auf Sichtbarkeit und Vergessen in der Medizingeschichte haben. Die Auseinandersetzung mit Dora Gersons Geschichte steht damit zugleich für eine kritisch-reflexive Erinnerungskultur innerhalb der Urologie.

## Abstract (en)

The history of urology is traditionally considered to be male-dominated. However, the field has never been exclusively “male medicine”—neither in terms of patients nor medical staff. This article highlights the life and work of Dresden-based physician Dora Gerson (1884–1941), one of the first German female specialists in urology and dermatovenereology, and draws attention to the widespread invisibility of women in the culture of memory of the field. Gerson studied medicine in Munich and Leipzig at the beginning of the 20th century and worked in both clinical and social medicine in the following years. In her Dresden practice, she combined urology, dermatology, and venereology, while also running a public counseling center for women with sexually transmitted diseases. In 1933, Gerson’s health insurance license was revoked, and she was forced to close her practice. From 1940 onward, she worked as a Jewish “medical practitioner” at the horticultural school in Ahlem, Hannover. In September 1941, under increasing pressure from repression, she took her own life. Her biography exemplifies a dual marginalization: as a member of a structurally disadvantaged gender and as a victim of Nazi persecution. The article connects Gerson’s life story with issues of gender, memory culture, and recognition practices in medicine. It demonstrates how professional cultural memory functions selectively and explores how social background, gender, and political circumstances influence visibility and oblivion in medical history. The reflection on Dora Gerson’s history thus also represents a critically reflective culture of remembrance within urology.

## Frauen in der Geschichte der Urologie – selten und ziemlich unsichtbar?

In den Statuten des 1865 in Leipzig gegründeten Allgemeinen Deutschen Frauenvereins heißt es, der Verein „hat die Aufgabe, für die erhöhte Bildung des weiblichen Geschlechts und die Befreiung der weiblichen Arbeit von allen ihrer Entfaltung entgegenstehenden Hindernissen mit vereinten Kräften zu wirken“ [1]. In einer Zeit fehlender weiblicher Bildungsmöglichkeiten (war für Frauen die höchste berufliche Qualifikation, die sie im 19. Jahrhundert erwerben konnten doch das Lehrerinnenexamen) ging es den Mitgliedern des Vereins um grundsätzliche Fragen der Chancengleichheit im Bildungsbereich, der freien Berufswahl und damit einhergehend um bessere Verdienstmöglichkeiten [2, 3].

Manifestiert hatte sich die hier zwischen den Zeilen herauslesbare Ungleichheit von Mann und Frau u. a. durch eine Entwicklung, die Mitte des 19. Jahrhunderts zum Abschluss gekommen war und als deren Ergebnis sich die Vorstellung etabliert hatte, dass „die Frau als ein dem normalen (männlichen) Menschen entgegengesetztes psychophysisches Sonderwesen“ [2] zu betrachten sei. Konsequenz dieses vermeintlich natürlichen Unterschieds war die geschlechtsspezifische Diskriminierung von Frauen, indem ihnen die Männer die Befähigung zu intellektuellen Berufen grundsätzlich absprachen. Noch in den 1880er-Jahren galt die „zerebrale Unterkapazität“ [4] von Frauen als wissenschaftlich ausgewiesenes Argument gegen ihre Zulassung zum Hochschulstudium [5]. Auch wenn sich, auf massiven Druck der deutschen Frauenbildungsbewegung, zwischen 1900 und 1909 die ersten Frauen im Deutschen Kaiserreich an der Universität immatrikulieren konnten [6], blieben alte Vorurteile und überholte Rollenklischees weiterhin existent.

So ließ sich auch in der akademisierten Medizin eine daran orientierte „Ordnung der Geschlechter in der Moderne“ beobachten [7]. Als geeigneter Ordnungsfaktor oder adäquates Differenzierungskriterium orientierte man sich in der Medizin v. a. an den Genitalien, der Psyche oder dem Skelett [8]. Dieses Narrativ kann auch die Geschichte der Urologie beschreiben, handelte es sich doch bei den Protagonisten der Professionalisierungsschichte (fast) ausschließlich um Männer.

Dabei ist die Urologie als medizinisches Fach nie eine reine „Männerheilkunde“ gewesen [9]. Dies macht beispielweise der seit jeher nicht unerhebliche Anteil an weiblichen urologischen Patienten deutlich. So führte Gustav Simon (1824–1876) im Jahr 1869 beispielsweise die weltweit erste Nephrektomie an der damals 46-jährigen Arbeiterin Margaretha Kleb aus Offenbach durch [10, 11]. Kleb überlebte und legte ihren Schnitt in einer der bekanntesten Illustrationen der Medizin frei [10, 12].

Somit darf es nicht verwundern, wenn uns in der frühen Professionalisierungsgeschichte der Urologie auch einige Medizinerinnen begegnen. Systematisch sichtbar wurden diese Frauen erstmals im Jahre 2009 im Zusammenhang mit der an der Medizinischen Hochschule Hannover von Jessica Peter verfassten Dissertation „Zur Geschichte der ersten Urologinnen in Deutschland“ [9]. Unter Anleitung des heutigen Archivars der Deutschen Gesellschaf für Urologie e.V. (DGU), Dirk Schultheiss, war hier eine wissenschaftliche Qualifikationsarbeit entstanden, die Kurzbiographien von frühen Medizinerinnen umfassten, die sich im Grenzgebiet zwischen Urologie, Dermatologie und Venerologie bewegen. Einige der dort aufgeführten Medizinerinnen wurden ebenfalls im Rahmen des Forschungsprojekts der DGU zur Geschichte der deutschen Urologen in der Zeit der Nationalsozialismus sowie allgemein zu verfolgten jüdischen MedizinerInnen im Nationalsozialismus noch einmal beleuchtet. Beispielhaft sind hier neben Dora Gerson (1884–1941) auch Johanna Hellmann (1890–1981) oder das erste weibliche DGU-Mitglied Dora Brücke-Teleky (1879–1963) zu nennen [6, 13–17].

Die DGU widmete sich auf dem Kongress 2022 in Hamburg diesen wichtigen Frauen im Rahmen der jährlichen wissenschaftshistorischen Ausstellung [17].

Aber obwohl die „Frauengeschichte“ oder die „Geschichte der Frau in der Medizin“ [2, 18–25] zunehmend Eingang in die medizinhistorische Forschung zur Geschichte der Urologie gefunden hat, wissen wir doch noch relativ wenig über die Pionierinnen dieses Fachs. Daher wird im vorliegenden Beitrag vor dem Hintergrund der Frauenemanzipation und des zu Anfang des 20. Jahrhunderts beginnenden Frauenstudiums das Lebens und Wirken der Urologin Dora Gerson (1884–1941) eingehend beleuchtet, einer der drei ersten Fachärztinnen für Urologie in Deutschland.

Zwar ehrt man innerhalb der Fachgesellschaft das Andenken der ersten deutschen Urologin Dora Teleky, nach der seit 2019 ein Preis der DGU benannt ist, weitere frühe Fachvertreterinnen profitierten bis heute jedoch nicht von den in den letzten Jahren eintretenden Konjunktur des Erinnerns an Pionierinnen in der Medizin [6, 17].

Nach einer Einführung zur (historischen) Debatte um vergeschlechtliche Zuschreibungen im Kontext wissenschaftlicher Qualifikation wird anhand der Vita der als jüdisch verfolgten Dora Gerson dargelegt, wo und wie Frauen in wissenschaftliche Erinnerungs- und Anerkennungsdiskursen der Urologie [26–29] eingehen – oder eben auch nicht. Gelten für Frauen eigene Regeln, um erfolgreich erinnert zu werden [30]? Und welchen Effekt hat das auf das fachkulturelle Gedächtnis [17, 29]?

## Der Weg zum Frauenstudium um 1900

Ein Blick zurück in die Geschichte zeigt, dass das Bild der Frau, je nach Epoche und Region, von unterschiedlichen gesellschaftlichen Normen geprägt war. Häufig kam ihr dabei eine eher untergeordnete Rolle zu. Folge dieser Marginalisierung war eine Bildungsungleichheit zwischen den Geschlechtern. So brachte auch die Schulreform in der ersten Hälfte des 19. Jahrhunderts für Mädchen und Frauen kaum Verbesserungen. Die ohnehin unterschiedlichen Ausgangsbedingungen für Jungen und Mädchen wurden noch verstärkt: „… was die Männer gründlich lernen, … erfahren unsere Mädchen ein klein wenig; das Wenige aber selten so, daß das Interesse für spätere Vertiefung rege gemacht oder das Selbstdenken ernsthaft in Anspruch genommen würde“ [31]. Die Bildung an den „höheren Töchterschulen“, die seit Beginn der allgemeinen Schulpflicht im Deutschen Reich im letzten Drittel des 19. Jahrhunderts allmählich entstanden, bedeutete lediglich die Vorbereitung auf die zukünftigen Aufgaben als Ehefrau und Mutter: „Alles was ein Frauenzimmer dem Staat für Dienste leisten kann, ist, daß es seinen Gatten zu seinen Geschäften aufheitert, gesunde Kinder gebieret, und sie zur Rechtschaffenheit und zu nützlichen Kenntnissen erziehet“ [32]. War es im 18. Jahrhundert einigen wenigen Frauen gelungen, sich durch Eigeninitiative auf akademische Prüfungen an Universitäten vorzubereiten (prominent darunter etwa die Ärztin Dorothea Christiane Erxleben), verhinderte die Implementierung des Abiturs als Voraussetzung für die Aufnahme eines Studiums nun auch diesen Weg. Im besten Fall endete der Bildungsweg von Schülerinnen mit dem Abschluss der höheren Töchterschule oder dem Besuch eines Lehrerinnenseminars. Somit war der Zugang zur universitären Bildung Frauen erschwert.

In den USA kam es bereits ab etwa 1840 zur Gründung von privaten Hochschulen für Frauen. Auch in England, genauer in Hitchin bei Cambridge, wurde 1869 das erste College for Women gegründet [33]. Im deutschen Sprachraum war die Schweiz führend: Bereits 1840 wurden in Zürich die ersten Gasthörerinnen zugelassen und Mitte der 1860er-Jahre, etwa zeitgleich mit Paris, wurden die ersten ordentlichen Studentinnen immatrikuliert. Österreich folgte 1897, wo die ersten Frauen als ordentliche Hörerinnen an der Philosophischen Fakultäten der Universität Wien zugelassen wurden [34].

Lag die Einzelfallentscheidung zur Zulassung von Frauen als Hörerinnen im Gebiet des Deutschen Reichs in den Anfängen noch bei den jeweiligen Dozenten, änderte sich dies rasch mit der steigenden Zahl von Interessentinnen und führte in den Folgejahren zu einer ablehnenden Haltung gegenüber den Gasthörerinnen. Erst in den 1890er-Jahren wurde das generelle Verbot wieder aufgehoben. Der Status als Gasthörerin berechtigte allerdings weiterhin in keinem der Gliedstaaten des Deutschen Reiches zur Teilnahme an einer akademischen Prüfung. Erst mit dem Bundesratsbeschluss von 1899 öffneten die ersten deutschen Universitäten ihre Pforten für weibliche Studierende. Voran gingen Heidelberg und Freiburg (Großherzogtum Baden), gefolgt von Bayern, Württemberg, Sachsen und Thüringen. 1908 schlossen sich Hessen, Elsaß-Lothringen sowie Preußen an. 1909 folgte Mecklenburg [35]. Nun waren Frauen in allen Teilen des Reichs grundsätzlich zum Studium zugelassen.

## Herkunft und Ausbildung Dora Gersons

**Abb. 1:**
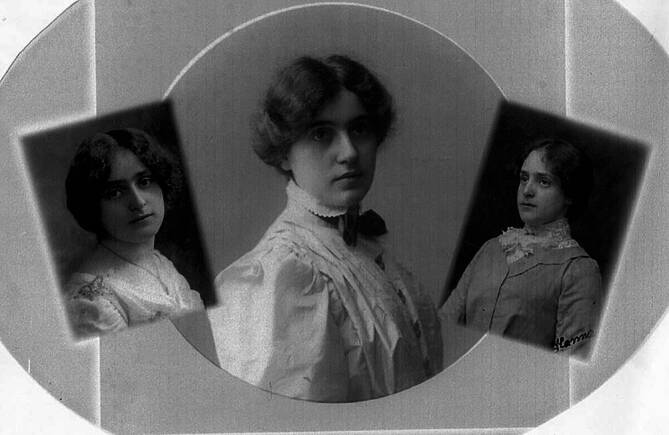
Die Schwestern Dora, Luise und Hanna Gerson (v. l. n. r.) um 1900 [38]

Dora Gerson kam am 24.09.1884 im sächsischen Aschersleben als zweitälteste Tochter des aus Soers stammenden jüdischen Bankiers Paul Gerson und seiner Ehefrau Ida Gerson (geb. Steinwehr) zur Welt^1^ [36]. Dora Gerson hatte insgesamt 5 Geschwister. Ihre älteste Schwester Johanna (Hanna; 1883–1951)^2^ war Künstlerin und heiratete den Schweizer Maler Albert Kohler (1883–1946; [37]), das Paar ließ sich nach der Eheschließung im schweizerischen Ascona nieder [36]. Die jüngste Schwester Luise (1886–1975)^3^ ehelichte in den 1920er-Jahren den Bankbeamten Friedrich Karl Paul Hagedorn. Ihr Bruder Rudolf Walter (1890–1939) wurde im Zuge des Novemberpogroms 1938 im KZ-Buchenwald interniert und ermordet. Die zwei weiteren Geschwister Eduard August (1882–1885)^4^ und Konrad (1888–1889)^5^ verstarben bereits im Kindesalter (Abb. 1).

Der Vater Paul Gerson zählte seit Mitte der 1880er-Jahre zum Mitinhaber der Ascherslebener Bank Gerson, Kohn und Co. und war einer der wenigen Millionäre der sonst eher für den Anbau von Majoran sowie ihren Kalibergbau und ihre Textilindustrie bekannten Region [39]. Er gehörte in den Jahren zwischen 1877 und 1884 der Synagogengemeinde zu Aschersleben an.^6^ Nicht genauer benannte politische Umstände veranlassten ihn jedoch dazu, mit seiner Glaubensgemeinschaft zu brechen und alle Mitglieder der Familie im Jahr 1898 in der Aschersleber Stadtkirche St. Stephani protestantisch taufen zu lassen [36]. Die Konversion der Familie fiel in eine Zeit, in der viele Personen jüdischen Glaubens von antijüdischen Ressentiments betroffen waren, welche sich im Laufe des 19. Jahrhunderts im Gewand eines neu aufkeimenden modernen Antisemitismus präsentierten. National-konservative Kreise glaubten in der gesellschaftlichen Integration und staatsrechtlichen Gleichstellung der Juden eine vermeintliche Überfremdung zu erblicken [36, 40]. Zwar stellte die Judenemanzipation nur eine von vielen politischen, sozialen und wirtschaftlichen Reformen des 19. Jahrhundert dar, doch führten die dadurch bedingten ökonomischen und rechtlichen Verbesserungen für die Juden de facto zu einer verstärkten, z. T. unterschwelligen, z. T. offenen gesellschaftlichen Ausgrenzung [41]. Dass ein jüdischer Arzt wie James Israel (1848–1926) 1894 eine außerordentliche Professur an der Berliner Universität erhalten konnte, obwohl er die Taufe abgelehnt hatte, stellte noch eine Seltenheit dar [42].

Wie Dora Gersons Lebenslauf aus ihrer Dissertation zu entnehmen ist, besuchte sie in Aschersleben für 8 Jahre die städtische Höhere Mädchenschule [43] sowie ein 2‑jähriges Privatinstitut in Kassel, was sie jedoch lediglich zur Erlangung eines Lehrerinnendiploms der Unterstufe befähigte. Um einen universitätsqualifizierenden schulischen Abschluss zu erhalten, führte ihr Weg im Anschluss an ein Mädchengymnasium in Karlsruhe. Die Stadt Karlsruhe bot bereits seit 1893 Gymnasialkurse für Schülerinnen und junge Frauen an, um sie auf ein Universitätsstudium vorbereiteten [44]. Neben Latein standen ebenfalls Mathematik und Naturwissenschaften auf dem Lehrplan [25, 44].

Ungeachtet der formalen Zugangsmöglichkeiten brauchten junge Frauen zu Beginn des 20. Jahrhunderts auch den finanziellen Hintergrund, um Zugang zu Bildung zu erhalten. Dies hatte zur Folge, dass „zwischen 1900 und 1930 gerade Jüdinnen des stark assimilierten, oft christlich konvertierten wohlhabenden jüdischen Bürgertums, ein Hochschulstudium … initiierte [n]“ [45]. Auch Dora Gerson entspricht diesem Sozialprofil und genoss über ihre Familie eine finanzielle Unabhängigkeit. So entsprach allein das am Karlsruher Mädchengymnasium vierteljährlich zu entrichtendem Schulgeld von 50 Mark [44] knapp dem Jahresgehalt eines Wirtschaftsfräuleins (210–400 Mark) oder einer Kindergärtnerin (240–420 Mark) zu dieser Zeit [46]. Auch die Aufwendung der Studiengebühren von ca. 80–230 Mark pro Semester gestaltete sich für die Familie Gerson als unproblematisch [13].^7^

Nach bestandener Reifeprüfung (1906) begann Dora Gerson 1906 zunächst ein Studium der Chemie an der Ludwig-Maximilians-Universität in München, um im Sommersemester 1907 zur Medizin zu wechseln.^8^ Nach erfolgreich abgelegter ärztlicher Vorprüfung (1908) führte der damals übliche Wechsel des Studienorts die Studentin an die heimatnahe Universität Leipzig (1908–1911).

Warum Gerson als ersten Studienort München wählte, lässt sich nicht definitiv sagen. Sie hätte sich im Wintersemestersemester 1906/1907 sowohl in Leipzig als auch in München als ordentliche Studentin immatrikulieren können.^9^ Vielleicht war es aber auch der renommierte Ruf der Münchener Medizinischen Fakultät, der sie zur Wahl des genannten Studienorts bewegte.

Bei Robert Rössle (1876–1956), der in den 1930er und 1940er-Jahren den Lehrstuhl für Pathologie an der Universität Berlin innehaben sollte, belegte Gerson im Sommersemester 1909 beispielsweise das pathologische histologisches Praktikum.^10^ Rössles Name ist bis heute Vielen auch außerhalb der medizinhistorischen Gemeinschaft ein Begriff, bildet die schwelende Auseinandersetzung um seine Verstrickungen in die Medizinverbrechen der NS-Zeit doch seit einigen Jahren die Grundlage einer Regen Debatte um die Umbenennung einer nach ihm benannten Straße in Berlin Pankow als Ausdruck eines reflexiven Geschichtsbewusstseins.^11^

Fest steht jedoch, dass sich mit Blick auf die Statistik die Ludwig-Maximilians-Universität bei Aspirantinnen des Medizinstudiums äußerster Beliebtheit erfreute [51].^12^ Im Sommersemester 1907 waren von den 302 regulär immatrikulierten Frauen an deutschen Universitäten rund 45 % angehende Ärztinnen. 46 von ihnen, also beinahe die Hälfte aller angehenden Medizinerinnen, studierten an der Medizinische Fakultät der Münchener Universität [48].

**Abb. 2:**
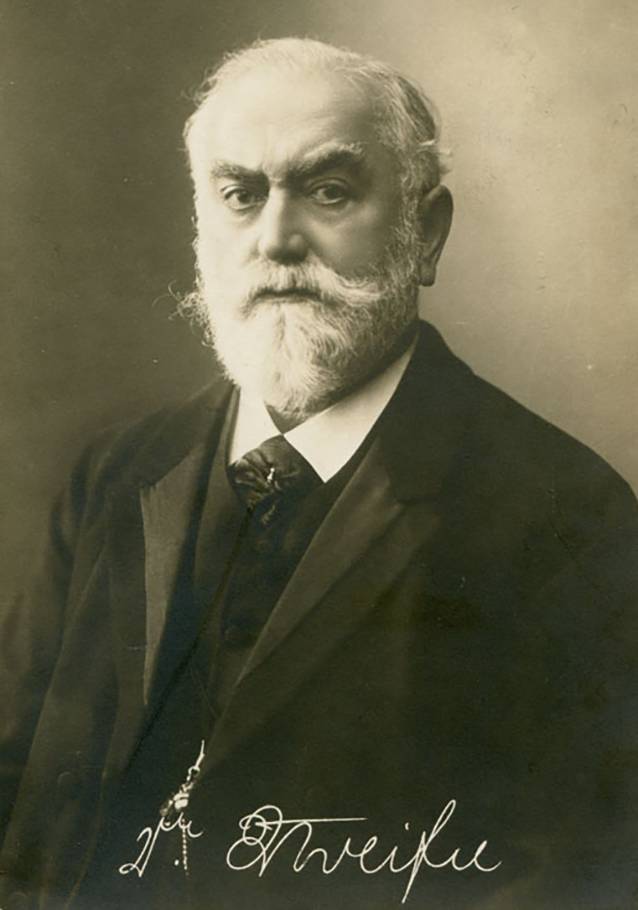
Paul Zweifel, um 1917 ©BY-SA 4.0. [57]

1909 wechselte Dora Gerson an die Universität in Leipzig, wo sie im Sommer 1911 das medizinische Staatsexamen bestand [9, 13]. Ihre Promotion verfasste sie im Grenzgebiet zwischen Urologie und Gynäkologie zum Thema „Über Uterusmyome als Indikation zu operativen Eingriffen während Schwangerschaft und Geburt“. 13Dora Gerson war damit eine von 40 Doktorandinnen, die zwischen 1902 und 1914 an der Medizinischen Fakultät der Universität Leipzig zum Dr. med. promoviert wurden [9, 48, 52].^14^ Wie bei der Majorität der an der Medizinischen Fakultät der Alma Mater Lipsiensis promovierten Doktorandinnen wird auch bei Gerson Paul Zweifel (1848–1927), der Leipziger Professor für Gynäkologie und Geburtshilfe, an erster Stelle der Referenten der Promotion erwähnt [9, 48, 53].^15^ Unter Zweifel (Abb. 2), der im Jahr 1887 die Nachfolge von Carl Siegmund Franz Credé angetreten hatte,^16^ avancierte Leipzig zu einem bedeutenden Zentrum der operativen Gynäkologie. Zudem gehörte Credé zu den frühen Verfechtern der Asepsis nach Semmelweis [52, 54–56].

**Abb. 3:**
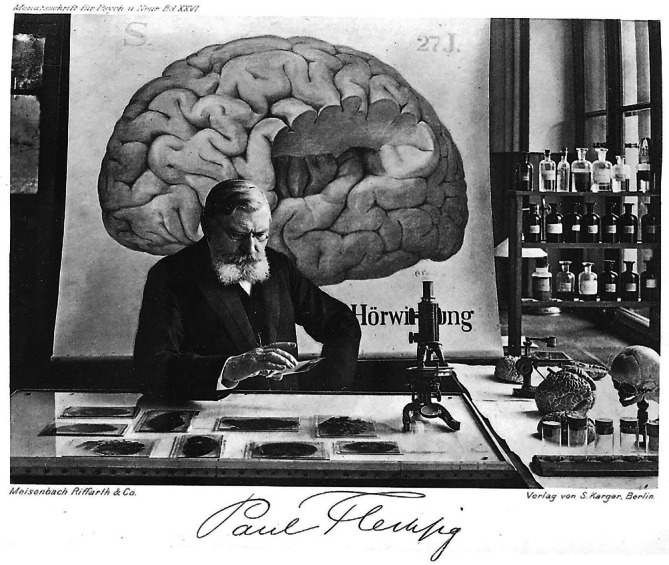
Paul Flechsig, um 1909 [58]

An zweiter Stelle der Referenten wird Paul Flechsig (1847– 1929) genannt (Abb. 3). Der Hirnforscher und Dekan der Medizinischen Fakultät Leipzig gehörte neben Zweifel und weiteren ordentlichen Professoren der Medizinischen Fakultät bereits vor dem offiziellen Immatrikulationsrecht für Frauen zu den frühen Promotoren desselben [52].

## Hinwendung zu Sozialhygiene und Venerologie

Im Anschluss an ihre Assistenzarztzeit (1912–1914) ging Dora Gerson an die Tuberkuloseabteilung des Augusta-Hospitals der Akademie für praktische Medizin Köln. Wissenschaftlich beschäftigte sie sich zu diesem Zeitpunkt v. a. mit sozialhygienischen Fragestellungen wie der Serodiagnosti der Tuberkulose [9, 15]. In den Jahren zwischen 1914 und 1919 wirkte Gerson dann als „Hilfsärztin“ am Städtischen Säuglingsheim Dresden sowie an der 1874 gegründeten II. äußeren Abteilung der demato-venerologischen Abteilung für Haut- und Geschlechtskrankheiten sowie kleine Chirurgie des Stadtkrankenhauses Dresden. In der Zeit vor der Einführung des Facharztes 1924 [59] wird hier ihre Spezialisierung in die Richtung der Venerologie sichtbar. Am Stadtkrankenhaus Dresden hatte Gerson 1919 und 1920 sogar eine Stelle als Oberärztin inne [9, 13–15, 60]. Der Erste Weltkrieg, in dem viele ihrer männlichen Kollegen Kriegsdienst leisteten, eröffnete ihr also Karriereoptionen.

Die Abteilung für Haut- und Geschlechtskrankheiten sowie kleine Chirurgie war zu dieser Zeit eine der wichtigsten Einrichtungen, die sich in Dresden auf die Behandlung von Geschlechtskrankheiten spezialisiert hatte. So lag der Ursprung des Departements in der Unterabteilung für syphilitische Frauen der äußeren (chirurgischen) Abteilung des Stadtkrankenhauses [61, 62]. Eine Ausweitung der Geschlechtskrankenversorgung stellte einen notwendigen Schritt dar, führte doch die Urbanisierung zu einer massiven Verbreitung sexuell übertragbarer Erkrankungen.

Im Kampf gegen Geschlechtskrankheiten bediente sich die Politik dabei des gesundheitspolitischen Instrumentariums der seit Ende des 19. Jahrhunderts aufstrebenden Sozialhygiene. Diese umfasste neben Aufklärung und Diagnose u. a. die Überwachung von sittlich gefährdeten Personen [14, 63, 64].

Das heißt, die oben genannten Maßnahmen gingen Hand in Hand mit der Bekämpfung der Prostitution. Ein Weg der Reglementierung bestand in Dresden wie auch andernorts in der polizeilichen Beaufsichtigung mittels Erfassung in Listen und regelmäßigen polizeiärztlichen Untersuchungen [65]. Diese Funktion übte Gerson neben ihrer klinischen Tätigkeit in dieser Zeit ebenfalls aus [66].

**Abb. 4:**
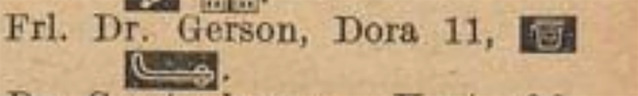
Ausschnitt Reichsmedizinalkalender 1926 [68]

Wie am Beispiel von Dora Gerson und dem Profil der II. äußeren Abteilung zu erkennen ist, bestand in den Zeiten vor der Bremer Richtlinie, der ersten deutschen Facharztordnung (1924), in Deutschland eine traditionell enge Verbindung zwischen den Fachdisziplinen Urologie, Venerologie und Dermatologie; eine Entwicklung, die mit der Etablierung der in der Richtlinie festgeschriebenen 14 „Sonderfächer“, darunter ebenfalls der Facharzt für Haut- und Geschlechtskrankheiten sowie für Erkrankungen der Harnorgane (Urologie), ihr Ende finden sollte [67]. „Eine vor 1924 häufig zu findende Bezeichnung des Spezialarztes für Haut- und Harnkrankheiten wurde in der Folge als nicht mehr zulässig erachtet“ [67]. Ungeachtet dessen fühlten sich Vertreterinnen und Vertreter dieses zuvor vergesellschafteten Sonderfachs auch nach dem Erlass der Bremer Richtlinie beiden Fachdisziplinen eng verbunden. So verwundert es auch nicht, dass Dora Gerson nach erfolgreicher Praxisgründung 1921 im Reichsmedizinalkalender (Abb. 4; [68]) als Fachärztin für Dermatologie und Venerologie sowie für Urologie firmierte.

Gerson gehörte damit zu den ersten Medizinerinnen jüdischer Herkunft, die sich in Dresden in eigener Praxis niederlassen sollten. Neben ihrer Praxis für Haut- und Geschlechtskrankheiten sowie Urologie bot sie ebenfalls eine kostenlose „Frauensprechstunde und Beratungsstelle für Geschlechtskranke“ in der Johannisstraße an. Im Sinne der oben erwähnten sozialmedizinischen Ausrichtung wurde in der Weimarer Republik für alle Patienten, die nicht über die finanziellen Mittel verfügten einen Arzt zu bezahlen, durch die Landesversicherungsanstalten Beratungsstellen für Geschlechtskranke eingerichtet. Diese Beratungsstellen wurden von Fachärzten geleitet. Wer sie aufsuchte, wurde unentgeltlich untersucht, beraten und dann einem Kassen- oder Privatarzt zur Behandlung überwiesen. Die Kostenübernahme erfolgte durch die Landesversicherungsanstalt selbst [65, 69]. Dora Gerson leitete dabei eine der insgesamt elf Beratungsstellen, die die Landesversicherungsanstalt in Sachsen unterhielt [65].

## Verfolgung im Nationalsozialismus

Obwohl Gerson schon im Kindesalter zum evangelischen Glauben konvertiert war, wurde sie im Nationalsozialismus als „Jüdin“ verfolgt.^17^ Als Konsequenz wurde sie am 01.04.1934 durch den privaten Krankenversicherungsverein Deutscher-Ring (PKV) von der Zulassung zur Rechnungserstattung durch die Krankenkassen ausgeschlossen [9, 13–15, 60, 66, 70]. Auch wenn es Gerson gelang, den Privatpraxisbetrieb noch bis 1935 aufrecht zu erhalten, zwangen sie die Umstände in der Folgezeit, ihre Praxis zu schließen [60, 70].

In 1936 erfolgte ein Umzug nach Hannover. Dort lebte zu dieser Zeit ihre Schwester Luise mit ihrem Ehemann. Luise überlebte den Zweiten Weltkrieg und die rassistische Verfolgung durch die Nationalsozialisten durch den Schutz und die Unterstützung eines jüdischen Verbindungsmannes. Dieser strich ihren Namen immer wieder aus den Deportationslisten für Theresienstadt [36]. Nachdem dieser Verbindungsmann jedoch selbst deportiert wurde, versteckte sich Luise bis zum Kriegsende im Untergrund. Ihr Mann, der seine Stellung als Reichsbeamter hatte niederlegen müssen, da er sich nicht von seiner „nichtarischen“ Ehefrau scheiden lassen wollte, sollte die finanzielle Existenz der Familie bis Frühjahr 1945 mit einem Tapetenhandel sichern [36].

Wie Dora Gersons fragmentarisch überliefertem Lebenslauf zu entnehmen ist, arbeitete sie nach ihrer Übersiedelung an der international renommierten „Israelitischen Gartenbauschule“ in Hannover-Ahlem als Hauswirtschaftsleiterin [9, 15, 60, 70, 71]. Hier sollte sie auch wieder zum jüdischen Glauben zurückkehren [70].

In der Zeit nach der nationalsozialistischen Machtübernahme avancierte die Gartenbauschule für viele als „nichtarisch“ Verfolgte zum Ort der Zuflucht und der Hoffnung. So engagierte sich die Schule für die Vorbereitung von Personen, die eine Auswanderung v. a. nach Palästina anstrebten [9, 70]. Diese Aufgabe sicherte nicht nur den Fortbestand der Institution, sondern bot zugleich Personal wie Schülern einen „Schutzraum auf Zeit“ [66, 70].

Ob Gerson ebenfalls Auswanderungspläne hegte, ist nicht belegt. Fest steht hingegen, dass gegen sie am 8. Februar1940 eine Sicherungsanordnung erlassen wurde [60, 70].^18^ Die Autorität dazu hatten seit dem „Gesetz zur Änderung des Gesetzes über die Devisenbewirtschaftung“ von 1936 die Devisenstellen („Stellen für Devisenbewirtschaftung“). Diesen oblag die fiskalische Überwachung und Ausbeutung der verfolgten jüdischen und jüdischstämmigen Bevölkerung. Zudem konnten im Kontext von „Ausbürgerungsverfahren“ die Devisenstellen auch Pässe konfiszieren oder Geldstrafen verhängen [72]. Eine solche Anordnung wurde immer dann erwogen, wenn der Verdacht einer Auswanderung bestand [72, 73]. Dies traf für Dora Gerson zu, lebte doch ihre älteste Schwester Johanna in der politisch neutralen Schweiz.

**Abb. 5:**
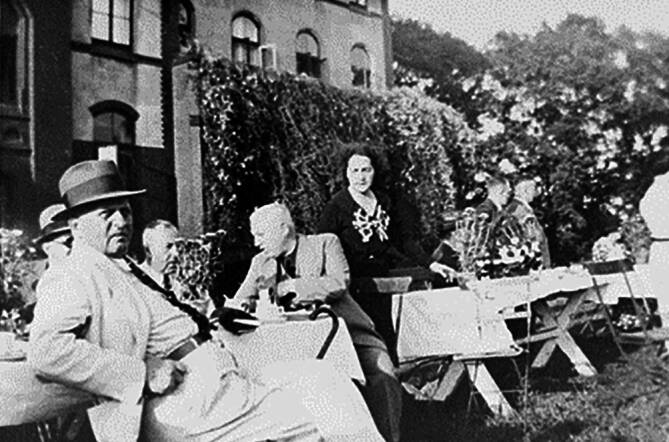
Dora Gerson (Mitte) vor dem Mädchenhaus, *links* neben ihr Direktor Leo Rosenblatt, ca. 1937, BaRH 84_175_008 (Nachlass Blumenthal)

Auch wenn bekannt war, dass Schweizer Grenzbeamte deutsche Flüchtlinge an der Grenze oftmals abwiesen, stellte eine Flucht ein durchaus mögliches Szenario in Gersons Fall dar [74]. Hinzu kam, dass sie nur einige Tage vor der Sicherungsanordnung, genauer am 3. Februar 1940, bei der Allgemeinen Deutschen Credit-Anstalt den Antrag gestellt hatte, über ihr komplettes Bankguthaben verfügen zu können.^19^ Im Zuge dieser Maßnahme entzog die Devisenstelle Dora Gerson die gänzliche Verfügungsgewalt über ihr Vermögen, um einen potenziellen Transfer ins Ausland zu verhindern [9, 70, 72, 75, 76].^20^ Dieser prekären Situation entkam sie nur durch die Mithilfe ihres Arbeitgebers. Der Direktor der Gartenbauschule Leo Rosenblatt bescheinigte ihr in der Folge nicht nur ihr Anstellungsverhältnis an der Gartenbauschule, sondern ebenfalls ein monatliches Bruttoeinkommen von lediglich 100 RM.^21^ Aufgrund dieses geringfügigen Verdienstes und der niedrigen Spareinlagen von 646 Reichsmark wurde die Sicherungsanordnung am 16. Februar wieder aufgehoben (Abb. 5).^22^

**Abb. 6:**
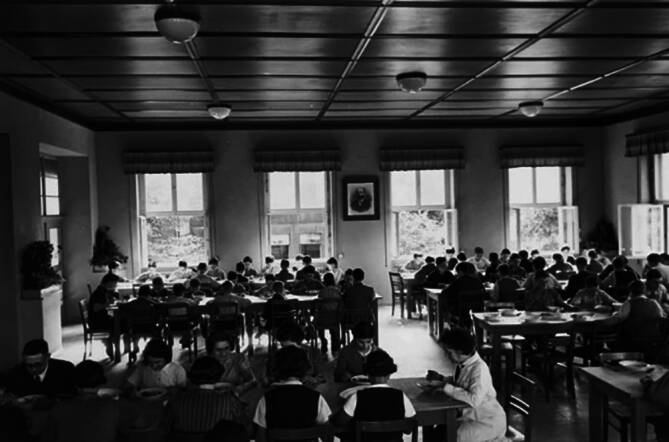
Dora Gerson (*vorn rechts* im weißen Kittel) im Speisesaal der Jüdischen Gartenbauschule Ahlem, 1938, Jüdisches Museum Berlin, Bestand Sonnenfeld, FOT 88/500/283/029 (mit freundl. Genehmigung)

Am 6. August 1940 erhielt Gerson die Zulassung als jüdische „Krankenbehandlerin“. Grundlage für diese Sondergenehmigung war die Absicherung der medizinischen Versorgung, der in der Gartenbauschule lebenden Kinder und Jugendlichen (Abb. 6; [77]). Sie war damit eine von 12 „Krankenbehandlern“, die für die ärztliche Versorgung von jüdischen Personen im Gau Süd-Hannover-Braunschweig zuständig sein sollte [36, 60, 66, 70, 77].

Was in den letzten 13 Monaten ihres Lebens geschah, ist nicht bekannt. Am 24. September 1941, ihrem 57 Geburtstag, nahm sich Dora Gerson das Leben [9, 15, 66, 70, 71, 78]. Die konkreten Gründe dafür liegen im Dunkeln, aber es ist nicht zu leugnen, dass die äußeren Umstände dazu beigetragen haben müssen, wie beispielsweise die reichsweite Einführung des Judensterns als sichtbares Zeichen der Stigmatisierung am 1. September 1941 [79]. Sven Eppinger charakterisiert diese äußeren Umstände mit den Worten „Klima der Angst“ und „Gefühl der Ausweglosigkeit der Lage“ [80]. In weiterem zeitlichem Zusammenhang mit Gersons Suizid steht ebenfalls die am 3. und 4. September 1941 durchgeführte „Aktion Lauterbacher“ [81], in welcher es auf dem Gelände der Gartenbauschule zu einer zwangsweisen Ghettoisierung von Hannoveraner Juden kam. Das dort entstandene Sammellager der Gestapoleitstelle Hannover sowie der Regierungsbezirke Hannover und Hildesheim diente der Vorbereitung für die Deportation der dort internierten Menschen [82]. Insgesamt wurden in der Zeit zwischen Oktober 1941 bis 1944 von dort aus 2173 Juden in die Ghettos Riga, Warschau, Theresienstadt und in das KZ Auschwitz deportiert [82]. Zudem wurde ab 1941 auf dem Gelände der Gartenbauschule auch ein „Judenhaus“ für jüdische Zwangsarbeit eingerichtet [82].

**Abb. 7:**
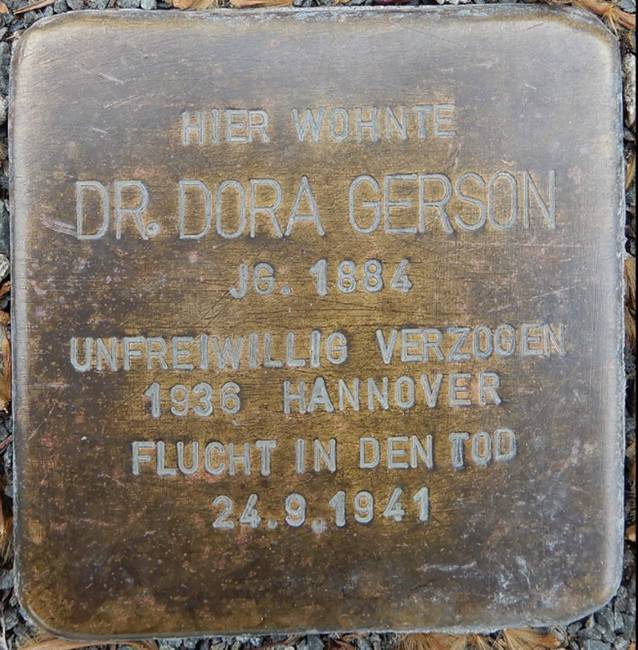
Stolperstein [83]

Ob auch Gerson in diesem Haus wohnen musste, lässt sich schlussendlich nicht belegen. Wie Jessica Peter bereits vor einigen Jahren resümierte, liegt dies jedoch nahe (Abb. 7; [9]).

Dora Gerson beging also zu einer Zeit Suizid, in der sich die (Über‑)Lebenssituation für die als Juden Verfolgten im nationalsozialistischen Deutschland noch einmal dramatisch zuspitzte. Eine Auswanderung erschien kaum mehr möglich und wurde kurz darauf, im Oktober, offiziell untersagt. Die „Flucht in den Tod“ muss vor dem geschilderten Hintergrund als letzter, selbstbestimmter Ausweg aus einer ausweglosen Situation verstanden werden [70].

## Fazit

Für die Mitte des 20. Jahrhunderts bemerkte die französische Pionierin des Feminismus Simone de Beauvoir: „Die Vorstellung der Welt ist, wie die Welt selbst, das Produkt der Männer: Sie beschreiben sie von ihrem Standpunkt aus, den sie mit dem der absoluten Wahrheit gleichsetzen“ [84]. Gilt dies in der fachkulturellen Erinnerung der Medizin im Allgemeinen und der Urologie im Besonderen bis heute? Woraus speist sich ein fachkulturelles Gedächtnis und welchen Einfluss hat dies auf die Entstehung einer Fachgeschichten vor dem Hintergrund einer selektiven Vergeschlechtlichung derselben?

Frauen waren bis Beginn des 20. Jahrhunderts in Deutschland vom Universitätsstudium ausgeschlossen, unter den Gründervätern der häufig um die Jahrhundertwende entstandenen medizinischen Fachgesellschaften findet man sie in der Regel nicht.

Der *Termicus technicus* des fachkulturellen Gedächtnisses nimmt die divergierenden Formen des Erinnerns in den (medizinischen) Wissenschaften, als Instrument bewusster Traditionsbildung, in den Blick. Als Zeugnisse dieser materiellen und immateriellen Erinnerungspolitik dienen Erinnerungsorte in „Wort und Schrift“ [28] wie beispielsweise Jubiläen, Nekrologe, Preise, Publikationen oder ebenfalls auch das Verlegen von Stolpersteinen und die Benennung von Straßen. Dabei unterliegt der Aushandlungsprozess des (nicht) Erinnert-Werdens einer ständigen Konjunktur und ist nicht frei vom Diktum einer hegemonialen Männlichkeit [17, 28, 29, 85].

Die Wiederentdeckung einer der ersten deutschen Urologinnen Dora Gerson erfolgte im Zuge einer kritischen Aufarbeitungsforschung der Medizin im Nationalsozialismus, wie die Arbeiten von Herrlich, Benzhöfer und Krischel darlegen [16, 86–88]. Ergänzt wurde dieses Erinnern durch Studien zur frauenemanzipatorischen Rolle früher (Fach‑)Ärztinnen in Deutschland [15], wie u. a. Jessica Peters Promotion zur Geschichte der ersten Urologinnen in Deutschland zeigt [9]. Mit den erwähnten Publikationen, insbesondere auch innerhalb der Urologie, wurde die Grundlage für eine erste Rezeption der Person Dora Gersons innerhalb und außerhalb der Geschichte der Urologie gelegt, die zuletzt zur Benennung der Dora-Gerson-Straße in Hannover, einer Straße auf dem Gelände des ehemaligen Oststadtkrankenhauses, geführt hat.

Ein genauer Blick auf Dora Gersons Biographie zeigt auch, aus welchen Gründen vielen anderen Frauen selbst nach der grundsätzlichen Öffnung der Universitäten der Weg zu einem Studium verschlossen blieb: Der Erwerb der allgemeinen Hochschulreife war allein aus finanziellen Gründen für viele junge Frauen ebenso undenkbar wie das Bezahlen von Studiengebühren – die natürlich auch junge Männer vor Herausforderungen stellten. Abseits einer kurzen Zeit während des Ersten Weltkriegs war selbst nach dem erfolgreichen Abschluss eines Medizinstudiums für Frauen eine Fortbildung nur in (unbezahlten) Volontärspositionen möglich. Für die Bankierstochter Dora Gerson waren diese Hürden überwindbar.

Heute machen Frauen etwa zwei Drittel der Medizinstudierenden und -absolventen aus [89]. Neben dem Geschlecht rücken aktuell auch die Einflüsse von Migrationshintergrund, sozialer Herkunft und Bildungsherkunft auf erfolgreiche Bildungsbiographien in den Blick, wobei positive und hemmende Einflüsse aus diesen Kategorien einander im Sinne der Intersektionalität noch verstärken können [51].

## Footnotes

1 Geburtsurkunde der Dora Gerson: Stadtarchiv Aschersleben, Standesamt-Aschersleben, Geburt 689/1886.

2 Geburtsurkunde der Johanna Gerson: Stadtarchiv Ascherleben-Standesamt Aschersleben, Geburt 604/1883.

3 Geburtsurkunde der Luise Gerson: Stadtarchiv Ascherleben-Standesamt Aschersleben, Geburt 476/1889.

4 Geburtsurkunde des Eduard August Gerson: Stadtarchiv Ascherleben-Standesamt Aschersleben, Geburt 547/1882.

5 Geburtsurkunde des Konrad Gerson: Stadtarchiv Ascherleben-Standesamt Aschersleben, Geburt 741/1888.

6 I/76/14 Gründung einer Synagogengemeinde.

7 UMA Stud-BB-288 (002); UMA Sud-BB-302 (002).

8 UAM, D‑V-52: Matrikel der im Studienjahr 1906/07 eingeschriebenen Studierenden.

9 Nach einem Entscheid des Prinzregenten Luitpold von Bayern war es seit dem Wintersemester 1903/04 an der Ludwig-Maximilians-Universität möglich, sich als Frau regulär zu immatrikulieren [47]. Leipzig folgte im April 1906 nach einer Verordnung des Sächsischen Kultusministeriums [48].

10 UAM, Stud-BB-316 = Sommersemester 1909.

11 Zur aktuellen Debatte siehe bitte [49, 50].

12 An der Münchener Universität absolvierte Margarethe Schiller aus Fürth bereits vor der offiziellen Zulassung von Frauen zum Hochschulstudium im Sommersemester 1903 ihr medizinisches Staatsexamen und konnte als erste Frau in Bayern zum Dr. med. promoviert werden [51].

13 UAL, Med. Fak. E 02/13, Bl. 72.

14 Ebd.

15 Ebd.

16 Credé gelang es beispielsweise, die Zahl der Neugeborenen, die Mitte des 19. Jahrhunderts an einer vom Erreger der Geschlechtskrankheit Gonorrhö verursachten Augenerkrankung litten, durch die prophylaktische Gabe von Augentropfen direkt nach der Geburt, drastisch zu senken. Darüber hinaus war er ein Verfechter der damals umstrittenen antiseptischen Maßnahmen der Händedesinfektion nach Ignaz Semmelweis [52].

17 ABEKM, MD, Rep. R03, Nr. 8320 1023/12, KB Aschersleben St. Stephani, Bild 26.

18 Nds. Landesarchiv, Abteilung Hannover: Bestand Hann. 210 Nr. Acc. 2004/025 Nr. 3656.

19 Nds. Landesarchiv, Abteilung Hannover: Bestand Hann. 210 Nr. Acc. 2004/025 Nr. 4270.

20 Nds. Landesarchiv, Abteilung Hannover: Bestand Hann. 210 Nr. Acc. 2004/025 Nr. 3656.

21 Nds. Landesarchiv, Abteilung Hannover: Bestand Hann .210 Nr. Acc. 2004/025 Nr. 4270. Die Medizinerin Peter wies in diesem Zusammenhang bereits darauf hin, dass das hier angeführte Gehalt im Vergleich zu dem durchschnittenen Jahresgehalt eines arischen Mediziners – 11.608 RM für das Jahr 1935 – als äußerst geringfügig eingestuft werden muss [9].

22 Nds. Landesarchiv, Abteilung Hannover: Bestand Hann. 210 Nr. Acc. 2004/025 Nr. 3656.
